# How Are New Vaccines Prioritized in Low-Income Countries? A Case Study of Human Papilloma Virus Vaccine and Pneumococcal Conjugate Vaccine in Uganda

**DOI:** 10.15171/ijhpm.2017.37

**Published:** 2017-04-08

**Authors:** Lauren Wallace, Lydia Kapirir

**Affiliations:** ^1^ Department of Anthropology, McMaster University, Hamilton, ON, Canada.; ^2^ Department of Health, Aging and Society, McMaster University, Hamilton, ON, Canada.

**Keywords:** Priority Setting, New Vaccines, Human Papilloma Virus (HPV) Vaccine, Pneumococcal Conjugate Vaccine (PCV), Low-Income Countries, Uganda

## Abstract

**Background:** To date, research on priority-setting for new vaccines has not adequately explored the influence of the global, national and sub-national levels of decision-making or contextual issues such as political pressure and stakeholder influence and power. Using Kapiriri and Martin’s conceptual framework, this paper evaluates priority setting for new vaccines in Uganda at national and sub-national levels, and considers how global priorities can influence country priorities. This study focuses on 2 specific vaccines, the human papilloma virus (HPV) vaccine and the pneumococcal conjugate vaccine (PCV).

**Methods:** This was a qualitative study that involved reviewing relevant Ugandan policy documents and media reports, as well as 54 key informant interviews at the global level and national and sub-national levels in Uganda. Kapiriri and Martin’s conceptual framework was used to evaluate the prioritization process.

**Results:** Priority setting for PCV and HPV was conducted by the Ministry of Health (MoH), which is considered to be a legitimate institution. While respondents described the priority setting process for PCV process as transparent, participatory, and guided by explicit relevant criteria and evidence, the prioritization of HPV was thought to have been less transparent and less participatory. Respondents reported that neither process was based on an explicit priority setting framework nor did it involve adequate representation from the districts (program implementers) or publicity. The priority setting process for both PCV and HPV was negatively affected by the larger political and economic context, which contributed to weak institutional capacity as well as power imbalances between development assistance partners and the MoH.

**Conclusion:** Priority setting in Uganda would be improved by strengthening institutional capacity and leadership and ensuring a transparent and participatory processes in which key stakeholders such as program implementers (the districts) and beneficiaries (the public) are involved. Kapiriri and Martin’s framework has the potential to guide priority setting evaluation efforts, however, evaluation should be built into the priority setting process a priori such that information on priority setting is gathered throughout the implementation cycle.

## Background

Vaccines are considered to be cost-effective health interventions, and play an important role in reducing under 5 mortality if high coverage is achieved.^1^ Building momentum from the Millennium Development Goals (MDGs), particularly those focusing on children, development assistance partners, such as the World Health Organization (WHO), United Nations International Children’s Emergency Fund (UNICEF), and Global Alliance for Vaccines and Immunization (GAVI) have become increasingly interested in and committed to facilitating the development of new vaccines and scaling up the implementation of existing vaccines. For example, in 2010, the Gates Foundation committed $10 million to vaccine research and the development and delivery of vaccines to the world’s poorest populations.^2^

Growing interest in vaccines as a useful health intervention has led to a “vaccine boom.” By the end of 2008, there were more than 120 vaccine related products developed – the highest in a century. However, not all vaccines that are developed are recommended for adoption. Since 2010, the WHO has recommended at least 6 new vaccines for adoption by low-income countries (LICs), these include pneumococcal conjugate (PCV), dengue, hepatitis A, influenza, rotavirus, and human papilloma virus (HPV) vaccines.^2^ Furthermore, there are about 25 vaccines in the research and development pipeline.^1^ These new vaccines, if adopted, have to compete for the meager public health resources within LICs, who are already responsible for the provision of at least 6 existing routine vaccines. Financial and human resources, as well as physical infrastructure, are necessary for the successful implementation of any vaccine program. The resource requirements for adopting new vaccines are greater than for existing vaccines. For instance, some new vaccines have protocols and cold chain requirements that are different from the routine vaccines and require retraining health workers and re-equipping health units.^3^ Prioritizing between new vaccines, while at the same time sustaining the existing vaccine program, is a major challenge for governments and Ministries of Health in LICs. Vaccine prioritization should occur through a clear and explicit process, as decisions to select one vaccine may occur at the cost of not investing in another and have consequences for health equity and outcomes.

Studies have described the decision-making processes involved in adopting new vaccines in LICs. For example, Burchett and colleagues conducted a study on the decision-making process for introducing vaccines in 12 LICs and identified 11 criteria that influence the adoption of new vaccines.^4^ Expanding on these issues, Mantel and Wang discuss the adoption of new vaccines in a commentary.^5^ While these studies provides insight into the prioritization of new vaccines, Burchett and colleagues identify a need for more detailed case studies that expand our understanding of the process. In particular, they note that more research is needed to explore larger contextual issues such as political pressure and stakeholder influence and power, and whether the implementation of vaccine priorities reflects official national-level objectives and processes.^4^ This information is needed to identify the most effective ways to improve decision-making, and ultimately, health outcomes.

This paper provides an in-depth exploration of priority setting for new vaccines in Uganda. The national and sub-national decision-making dynamics in the Ugandan public health system are similar to those in other LICs, and thus, provide a basis for generalizations beyond the Ugandan context. Based on the critical role that donors play in the adoption of new vaccines,^3^ this paper also considers how global priorities can influence country priorities. This study evaluates priority setting for vaccines in Uganda using Kapiriri and Martin’s conceptual framework for evaluating priority setting.^6^ This analysis is based on two specific vaccines, the HPV vaccine and the PCV.

## Methods

### The Analytical Framework

Kapiriri and Martin’s framework was developed in 2010 and is based on the priority setting literature and Delphi interviews with researchers involved in health prioritization research in low- and middle-income countries (LMICs). In 2015, the framework was validated by researchers and policy-makers in both global and LMIC contexts (Forthcoming), making it particularly relevant for an examination of priority setting for new vaccines in Uganda. The framework is holistic since it addresses the potential roles played by the social, cultural, political and economic context, which cannot be overlooked when discussing priority setting in LMICs.

Kapiriri and Martin’s framework identifies 23 major parameters for successful priority setting (see Table 1).^6^ Each parameter is organized according to whether they are (1) internal/external relative to the priority setting institution, and (2) immediate/delayed; whereby immediate measures are expected within a planning/fiscal/budget year and delayed measures are expected beyond 3 fiscal years. Each parameter is associated with objectively verifiable indicator(s) (OVI) and means of verification (MOV). These specify the evidence that will inform evaluators if an expected measure has been achieved. By being objectively verifiable, these indicators permit different people, using the same measuring process, to obtain the same results independently.^7^

### Data Collection

This is a qualitative study involving a review of documents and key informant interviews.

Data collection strategies were aligned with the MOV proposed in the framework, where possible.

### Document Reviews

Policy documents and media reports were reviewed. Policy documents include the health sector strategic and investment plans (HSSIPs) and the Uganda National Expanded Program on Immunization’s (UNEPI’s) comprehensive multiyear plans for immunization (CMYPs) between 2000-2015.^8-13^ While the HSSIPs describe the country’s priorities for new vaccine adoption, the CMYPs show what is actually implemented.

Media reports on HPV and PCV published within the same period were also reviewed.

Media reports provide details of the rationale for the priorities set, and the process of rolling out vaccines to the public. Media reports also showcase public awareness of the priority setting process; the public is a key stakeholder in vaccine priority setting and new vaccine introduction requires intensive communication and social mobilization targeting communities to provide information about new vaccines.

### Interviews

Interviews were conducted between 2013 and 2015. Respondents were identified from the 3 main levels of priority setting – the global, national and sub-national (district levels). Fifty-four interviews were conducted in total (see Table 2). Global level respondents provided context for understanding how global level priority setting impacts national level priority setting. National level respondents described the priority setting process within Uganda. Follow-up interviews with national actors provided information on which priorities were implemented and why. District level participants were critical since, according to the national health policy, district managers are responsible for policy implementation in Uganda. These participants provided insight into the enablers and barriers to policy implementation.

### Study Sample and Sampling Strategy

Purposive sampling was used, whereby only people deemed to be knowledgeable of, or involved in, new vaccine introduction were recruited. Respondents at the global level were identified through their organization’s web page. Within Uganda, the initial respondent was the officer in charge of the Expanded Program on Immunization (EPI); he identified subsequent respondents. Three districts were selected to reflect diverse settings, (ie, regional – North, Central and East, rural/urban, and new/old districts).

The interviews were conducted by the principal investigator (LK) and trained Ugandan research assistants, using a pilot-tested interview guide. Global level interviews were conducted by telephone while the Ministry of Health (MoH) and district interviews were conducted face to face. Interviews lasted an average of 45 minutes and were audio recorded with permission from the respondents.

### Data Analysis

Interviews were transcribed verbatim and data were analyzed using Nvivo 10. Initially, 3 members of the research team independently read and coded two interviews, developing a list of codes. They then convened to discuss the code names and any discrepancies and to develop an agreed upon code list. Similar codes were grouped into categories and related categories linked to overarching themes. Further analysis involved using Kapiriri and Martin’s framework^6^ to assess the degree to which the described process met the parameters for successful priority setting (summarized in Table 1).^6^

**Table 1:** Parameters for Evaluating Priority Setting With Corresponding Means of Verification and Indicators

**Immediate Parameters of Successful Priority Setting**	**OVIs**	**MOV**
Efficiency of the priority-setting process	Proportion of meeting time spent on priority setting, number of decisions made on time	Observations/minutes at meetings, annual budget documents, health system reports
Allocation of resources according to priorities	Degree of alignment of resource allocation and agreed upon priorities, times budget is re-allocated from less prioritized to high prioritized areas, stakeholder satisfaction with the decisions	Annual budget reports, evaluation documents
Stakeholder participation	Number stakeholder participating, number of opportunities each stakeholder gets to express opinion	Observations/minutes at meetings, media reports, special reports
Use of clear priority setting process/tool/method	Documented priority setting process and/or use of priority setting framework	Observation/minutes at meetings, media reports, special reports
Use of evidence	Number of times available data is resourced/number of studies commissioned/existing strategies to collect relevant data	Observations/minutes at meetings, media reports, special reports
Use of explicit relevant priority setting criteria	Documented/articulated criteria	Observations/minutes at meetings, media reports, special reports
Publicity of priorities and criteria	Number of times decisions and rationales appear in public documents	Media reports
Functional mechanisms for appealing the decisions	Number of decisions appealed, number of decisions revised	Observations/minutes at meetings, media reports, special reports
Functional mechanisms for enforcement	Number of cases of failure to adhere to priority-setting process reported	Observations/minutes at meetings, media reports, special reports
Fairer priority setting process a) relevance b) publicity c) revisions d) enforcement	Degree to which priority setting process aligns with 4 conditions of Accountability for reasonableness: relevance-involvement of relevant stakeholders and relevant criteria, publicity of decisions, mechanisms for revisions and enforcement of priority setting process	Observations/minutes at meetings, media reports, special reports
Reflection of public values	Number and type of members from the general public represented, how they are selected, number of times they get to express their opinion, proportion of decisions reflecting public values, documented strategy to enlist public values, number of studies commissioned to elicit public values	Observations/minutes at meetings, study reports, meeting minutes and strategic plans
Increased public awareness of priority setting	% of public aware of existing priority setting process	Public awareness study
Increased public confidence in and acceptance of decisions	Number of complaints from the public	Reports, minutes from meetings, media reports
**Delayed Parameters of Successful priority setting**		
Increased stakeholder understanding, satisfaction and compliance with the priority setting process	Number of stakeholder attending meetings, number of complaints from stakeholder, % stakeholder that can articulate the concepts used in priority setting and appreciate the need for priority setting	Observations/minutes at meetings, special reports, stakeholder satisfaction survey, media reports, stakeholder interviews, evaluation reports
Decreased dissentions	Number of complaints from stakeholder	Meeting minutes, media reports
Decreased resource wastage	Proportion of budget unused	Budget documents, evaluation reports
Improved internal accountability/reduced corruption	Number of publicized resource allocation decisions	Evaluation reports, stakeholder interviews, media reports
Strengthening of the priority setting institution	Indicators relating to increased efficiency, use of data, quality of decisions and appropriate resource allocation, % stakeholders with the capacity to set priorities	Training reports, evaluation reports, budget documents
Impact on institutional goals and objectives	% of institutional objectives met that are attributed to the priority setting process	Evaluation reports, special studies
Impact on health policy and practice	Changes in health policy to reflect identified priorities	Policy documents
Achievement of health system goals	% Reduction in DALYs, % reduction of the gap between the lower and upper quintiles, % of poor populations spending more than 50% of their income on health care, % users who report satisfaction with the health care system	National budget allocation documents, human resources survey reports, Interviews with stakeholders
Improved financial and political accountability	Number of publicized financial resource allocation decisions, number of corruption instances reported, % of the public reporting satisfaction with the process	Reports, media reports, interviews with stakeholders
Increased investment in the health sector and strengthening of the health care system	Proportion increase in the health budget, proportion increase in the retention of health workers, % of the public reporting satisfaction with the health care system	

Abbreviations: OVIs, objectively verifiable indicators; MOV, means of verification; DALY, disability-adjusted life year.

**Table 2:** Study Participants by Level of Decision-Making

**Level of Decision-Making**	**Number of Respondents**
District	27
MoH	19
Global level	8
Total	54

Abbreviation: MoH, Ministry of Health.

## Results

### Documented Priority Setting Processes for New Vaccines at the Global Level

#### World Health Organization

According to respondents, vaccines prioritized at the national level are chosen from a global menu of approved vaccines. This menu is created from a list of recommendations put forward by the WHO. Once a new vaccine product is developed and presented to the WHO, WHO seeks advice from its Strategic Advisory Group of Experts (SAGE). SAGE convenes advisory working groups with the relevant expertise to synthesize evidence and produce a position paper on new vaccines, which is publicized at the global, regional and national levels. SAGE provides recommendations to WHO, who present their recommendations to the World Health Assembly. This information filters down to the WHO in country and regional offices, who encourage countries to adopt the new vaccines. The criteria used by WHO for vaccine prioritization include: demand in the respective UN-supplied markets, WHO compliance needs with international health regulations, eradication, elimination or control initiatives and immunization program considerations, and finally, SAGE recommendations and the security/sustainability of supplies.^14^ Respondents described how WHO plays an important role in the development of the new vaccine menu. For example, one individual argued that: “.*..there’s a lot of trust in the WHO team. By the time they heavily suggest an introduction of a vaccine it’s usually pretty much gospel truth…”* [G_8]*.*

#### Global Vaccine Alliance

While new vaccines may be recommended by WHO, the board of the GAVI independently approves the menu of vaccines that will be subsidized. WHO and GAVI priority vaccines are summarized in Table 3. GAVI undertakes a very systematic and rigorous process called a vaccine investment strategy every 2 to 3 years to determine which vaccines to support. Two of the most important criteria that GAVI uses to determine which vaccines to prioritize are cost-effectiveness and burden of disease. Once the menu has been finalized, GAVI calls for proposals from countries that are eligible for GAVI subsidization. Only LICs with a Gross National Product of less than $1580 are supported by GAVI.^15^

**Table 3:** Vaccines Recommended by WHO and Those Funded by GAVI

**Priority Vaccines**	**WHO**	**GAVI**
Cholera (oral)	x	x
DTwP-based combination containing IPV (DTwP-Hep B-Hib-IPV)	x	-
Japanese encephalitis	-	x
Dengue	x	-
Hepatitis A	x	-
HPV	x	x
Malaria	x	-
Measles-rubella	x	-
MMR	x	-
Pneumococcal conjugate	x	x
Polio (inactivated) [IPV]	x	-
Polio (bivalent live oral) (bOPV1,3)	x	-
Rotavirus	x	x
Typhoid conjugate	x	-
Yellow fever	x	x
Meningitis A	-	x
Pentavalent vaccine	-	x

Abbreviations: WHO, World Health Organization; GAVI‏, Global Alliance for Vaccines and Immunization; MMR, measles-mumps-rubella; HPV, Human Papilloma Virus; IPV, inactivated polio vaccine. Source‏: http://www.who.int/immunization_standards/vaccine_quality/pq_priorities/en/; http://www.Gavi.org/support/nvs‏/

GAVI eligible countries must submit their proposals for vaccine introduction through country multi-year plans. After the submission of the proposal to GAVI, an assessment of the in-country capacity of the cold chain system and other logistics, known as an Effective Vaccine Management Assessment (EVMA) is conducted. This is followed by an Effective Vaccine Management Improvement Plan to improve the health system’s capacity to manage the new vaccine. This evidence is fed into the country’s GAVI proposals to indicate the country’s ability to sustain the introduction of the vaccine.

GAVI’s new vaccine introduction support consists of providing eligible countries with a substantially subsidized supply of the vaccine, as well as a one-time grant to assist with the initial expenses of vaccine introduction. The grant does not cover the full costs of new vaccine introduction, but rather works ‘‘to facilitate the timely and effective implementation of critical activities in the national vaccine introduction plan in advance of a new vaccine introduction and cover a share of the pre-introduction activities.”^15^

Multiple respondents described how GAVI affects the prioritization process at both the global and national levels, by influencing the choice of vaccines included on the new vaccine menu and through directly funding specific vaccines.

*“…the country is not allowed to get other vaccines partly because of the GAVI contract…”* [G_8].

*“...So yea if GAVI puts for us a menu of vaccines that they’re subsidizing it also kind of affects the priority setting process...”* [G_4].

GAVI approves the menu of vaccines that can be subsidized in LICs. However, vaccines adopted are approved through extensive and participatory national level processes. These national level processes are described below.

#### Documented Priority Setting Processes for New Vaccines at the National Level

According to respondents, priority setting for new vaccines in Uganda is part of the national level priority setting process which involves the development of the HSSIPs, where 5-year national MoH priorities are identified by a broad range of stakeholders including government officers, development assistance partners, civil society organizations, and representatives from the districts. According to these reports, immunization and introduction of new vaccines have been consistently identified among the national priorities.^8-10^ The 5-year plans specifically identify the new vaccines that should be introduced during the 5-year period. These decisions are guided by evidence presented by the medical officer in charge of the EPI, which has been derived from prior processes that are focused specifically on prioritization of new vaccines (described below). National level priorities are then presented to the Health Interagency Policy Advisory Committee (HIPAC), which gives the final approval and monitors vaccine implementation.

#### Processes for New Vaccine Prioritization

The prioritization process for new vaccines involves different institutions and stakeholders namely: the Uganda National Academy of Science (UNAS), Uganda National Immunization Technical Advisory Group (UNITAG), or the Advisory Committee on Vaccines and Immunization (ACVI), the National EPI, the Maternal and Child Health Technical Working Group (TWG) and the HIPAC. The process is often triggered when the WHO and/or GAVI recommends a new vaccine. UNAS then takes the responsibility of electing a relevant advisory committee for the new vaccine, referred to as the NITAG or the ACVI. The composition and roles of these different institutions and committees are summarized in Table 4.

**Table 4:** Documented Stakeholders and Institutions Involved in Priority Setting for New Vaccines in Uganda by Level of Decision-Making

**Organization/Institution**	**Level of Decision-Making**	**Stakeholders Involved**	**Roles**
HIPAC, GAVI, UNICEF, WHO, USAID, JICA, PATH, AFENET	Global, National	Development assistance partners	Funding, Technical and logistic support
MoH General	National	Government officers (National and sub-national)	Policy direction for prioritizing and monitoring new vaccines
MoH-EPI program and the Maternal Health Technical Working Group	National	Government officers, representatives from the civil society and the academia	Develops policy documents and recommendations to the MoH; and CMYPs for GAVI
UNAS and UNITAG	National	Government officers, Academics, Pediatricians, Vaccinologists, Civil Society, Economists, Politicians, WHO health systems representative, technical advisors	Technical support to provide objective and evidence based recommendations to the MoH for new vaccines
District	Sub-national	Local government officers	Implement routine and new vaccine regimens
Public	National and sub-national levels	Public	Provide input on acceptability of new vaccines

Abbreviations: MoH‏, Ministry of Health; WHO, World Health Organization; UNICEF, United Nations International Children’s Emergency Fund; GAVI, Global Alliance for Vaccines and Immunization; USAID, United States Agency for International Development; HIPAC, Health Interagency Policy Advisory Committee; EPI, Expanded Program on Immunization; UNAS, Uganda National Academy of Science; UNITAG; Uganda National Immunization Technical Advisory Group; CMYPs, comprehensive multiyear plans for immunization; JICA, Japan International Co-operation Agency; AFENET, African Field Epidemiology Network.

The UNAS has developed clear criteria for identifying members of the NITAG and explicit guidelines that the NITAG should use when evaluating the introduction of new vaccines. The NITAG collects and reviews evidence on the potential new vaccines and makes evidence-based recommendations to the UNAS. Based on these recommendations, the UNAS advises the TWG within the MoH, (chaired by the EPI manager). Once the TWG makes the decision to recommend the introduction of a new vaccine, the EPI manager develops a proposal and presents it to the senior management committee in the MoH; once approved this proposal is submitted to HIPAC. If Uganda were able to fund the introduction of the new vaccines, the next step would have been for the national medical stores to purchase the approved vaccines. However, since the country cannot afford the costs of new vaccines, they have to apply for funding from GAVI. Therefore, after the government structures have approved the introduction of a new vaccine – a critical condition for GAVI funding – the EPI manager includes those new vaccines in the proposal for GAVI funding.

If a new vaccine is approved by GAVI, the country is funded to introduce the vaccine. However, before the funding is approved, GAVI conducts an in country EVMA, to establish the country’s readiness to introduce the new vaccine. Next, in country stakeholders utilize feedback from the GAVI secretariat to prepare an effective vaccine management improvement plan to ensure that the country is ready to implement the new vaccine. This may involve establishing in country surveillance to establish baseline indicators at introduction, cold chain preparation, waste management preparation, editing data management forms, health worker training, communication and community mobilisation. Once the recommendations have been implemented, GAVI releases the funds to facilitate the introduction of the new vaccine. Vaccines are then procured at the national level and delivered to the districts. While the districts are partly in charge of implementation, the EPI and the MoH are in charge of disease surveillance and reporting of adverse effects. The existing process has been described above. However, this process is not always followed, as was exemplified in the cases of introducing PCV and HPV.

#### Priority Setting for New Vaccines in Uganda: The Cases of PCV and HPV

This section is organized according to the parameters of successful priority setting that could be evaluated (summarized in Table 5). It was not possible to evaluate some of the parameters namely: efficiency of the priority setting process, reflection of public values, increased public awareness of priority setting, increased public confidence in and acceptance of decisions, and decreased resource wastage.

**Table 5:** Evaluating the Introduction of HPV and PCV Using the Parameters of Successful Priority Setting

**Immediate Parameter of Successful Priority Setting**	**HPV**	**PCV**
Efficiency of the priority-setting process	Impossible to determine	Impossible to determine
Allocation of resources according to priorities	Although identified as a priority, HPV was not originally identified as a high priority in HSSIP II.	Identified as a priority in the HSSIP II.
Stakeholder participation	Participation of key stakeholders such as MoH staff and district officers limited	Same as HPV
Use of clear priority setting process/tool/methods	No explicit framework or process used	No explicit framework or process used
Use of Evidence	Evidence from pilot project in 2 districts and several feasibility studies used	Evidence from sentinel surveillance reports and commissioned studies used
Use of explicit and relevant priority setting criteria	Burden of disease, equity used	Burden of disease and cost-effectiveness used
Publicity of priorities and criteria	Media reports about benefits of vaccines, launching of vaccines, however public not informed of exact decision-making processes	Same as HPV
Functional mechanisms for appealing the decisions	None recorded	Same as HPV
Functional mechanisms for enforcement	None recorded	Same as HPV
Fairer priority setting process (*a*) relevance (*b*) publicity (*c*) revisions (*d*) enforcement	Less fair than for PCV	Fairer than for HPV
Reflection of public values	Impossible to determine	Same as HPV
Increased public awareness of priority setting	Impossible to determine	Same as HPV
Increased public confidence in and acceptance of decisions	Impossible to determine	Same as HPV
**Delayed Parameter of Successful Priority Setting**		
Increased stakeholder understanding, satisfaction and compliance with the priority setting process		
(*a*) Stakeholder understanding	Limited understanding especially at district level	Clearer since they followed due process of implementing HSSIP identified priorities
(*b*) Stakeholder satisfaction	Dissatisfaction with the introduction of HPV when it was not part of the original plan	General satisfaction since PCV was part of the original HSSIP plan
(*c*) Stakeholder compliance	Sense that compliance had deteriorated, especially that of donors	Same as HPV
Decreased dissentions	Other than media reports of complaints related to vaccines running out in districts, no complaints recorded	Same as HPV
Decreased resource wastage	Impossible to determine	Same as HPV
Improved internal accountability/reduced corruption	Institutional transparency low since criteria for prioritization thought to be irrelevant	Transparency better but still lacking because of lack of consultative process
Strengthening of the priority setting institution	See strengthening of the healthcare system	See strengthening of the health care system
Impact on institutional goals and objectives	See achievement of health system goals	See achievement of health system goals
Impact on health policy and practice	No impact on health policy but impact on practice since changes in vaccine schedule	Same as HPV
Achievement of health system goals	Contributed to goal of reducing mortality and morbidity	Same as HPV
Improved financial and political accountability	Financial accountability appears to be met for HPV	Respondents reported one instance where the resources where misappropriated but it was rectified. More stringent accountability mechanisms have been instituted
Increased investment in the health sector and strengthening of the health care system	Contextual issues weakened capacity to successfully engage in priority setting	Same as HPV

Abbreviations‏: HPV, Human Papilloma Virus; PCV, pneumococcal conjugate vaccine; HSSIP, Health Sector Strategic and Investment Plan; MoH, Ministry of Health.

#### Allocation of Resources According to Priorities

According to this parameter, resource allocation should be based on the priorities that are identified. While the decision to introduce PCV was deemed appropriate by our respondents, several thought that the allocation of resources to HPV in 2005-2010 and 2010-2015 in the CMYPs was inappropriate since this did not match the new vaccines that were prioritized in the HSSIPs. For instance, in HSSIP III,^8^ the focus was on yellow fever and hepatitis B, yet resources were allocated to the introduction of HPV, in addition to hepatitis B and rotavirus.^12^

#### Stakeholder Participation

This parameter relates to whether relevant stakeholders were involved in the priority setting process and were able to meaningfully contribute to the process. While the documents described a participatory process and explicit participation structures, our interviews revealed that not all the relevant stakeholders were actually involved in the process and that some stakeholders were involved in an inappropriate way. Stakeholders who were not adequately involved in the priority setting process include: MoH officers, district officers and the public. According to respondents, development assistance partners were involved in the priority setting process an inappropriate way. The participation of these stakeholders is discussed in detail below.

#### Development Assistance Partners

In participants’ discussions of priority setting for PCV and HPV, there was consistent reference to the role that development assistance partners played in the introduction of the new vaccines. In some cases, development assistance partners priorities seemed to trump considerations of explicit criteria. Participants discussed the way that development assistance partners’ influence led original vaccine priorities stated in the HSSIPs to be rearranged. For example, a policymaker from the MoH argued that the MoH’s decision to prioritize PCV was based on sound evidence; however, donor influence may have influenced the early introduction of HPV.

*“…I’ll give an example, within the priorities that were set, what we [the MOH] had agreed was that a cost effective study was done and it was clear that if we introduced pneumococcus vaccine [first] it’s more cost effective. Now somebody comes up and gives a donation of HPV vaccine to the government…With that development, the priorities seem to change because now somebody has come with their agenda [to push for their] vaccine. And it looks like a tempting offer… So at the end of the day it’s like reversing [the HSSIP priorities] to start with HPV then PCV”* [U_1].

There was a sense that development assistance partners’ adherence to a consultative priority setting process had decreased. Study participants who are members of development assistance partners argued that development assistance partners dominated forums for harmonizing partners, such as the Immunization Inter-Agency Coordinating Committee (IACC). According to one respondent, contrary to its intended role, the IACC had become “an opportunity for each partner to come with his influence” and to “push in a specific direction” and “the government has very little say in this type of forum” [G_7].

#### Ministry of Health Officers

While participants reported that the involvement of development assistance partners were uneven, they also alluded to 2 special categories of stakeholders – MoH officers and districts – who they thought were critical to the implementation of the new vaccine program, but were insufficiently involved in setting priorities for vaccines. There were sentiments that there was limited involvement of the MoH officers in the development of HSSIP III since at the time, the MoH was only at 10% of its staffing levels. This plan was reportedly developed mainly through outsourcing. One Ugandan respondent working for a development assistance partner organization argued that while in the beginning stakeholders at all levels of government (including districts) participated in the priority setting process, with time, the process became less consultative and transparent, with only a handful of actors from the MoH and development assistance partners driving the process.

“…*At the beginning the [priority setting] process was transparent and it was very consultative…We had excessive discussion with local leaders from the district, [and at the] national level, [we even had discussions with] line ministries, public service and finance…our partners both at the national level and sub national level…But over time the Ministry leadership has somehow weakened so the consultative [priority-setting] process has died down. Now it is prescriptive…the MoH comes up with priorities and they’re given to districts to implement... So the process has now been restricted to a few individuals [at the MoH] and the donors taking the upper hand”* [U_ 20].

#### District Officers

Respondents reported a disconnect between national initiatives, such as the introduction of new vaccines, and processes within the districts. Participants argued that in many cases the districts are neither consulted during the priority setting process, nor made aware of the national vaccine priorities. This results in the districts failing to implement the vaccines since they have not put strategies in place to operationalize them. For example, 2 participants argued:

*“…Currently I think the link [between the national and district priority setting] is very poor*”

[U_2].

*“[The districts] didn’t have an annual work plan. So I don’t know what they’re implementing. So implementation is not being guided by priorities in the district”* [U_10].

District-level respondents argued that they had not been given adequate opportunities to participate in priority setting discussions at the national level; they suggested that this was problematic since only individuals working at the district level could truly understand the specific needs of their population, and the feasibility of proposed interventions.

*“We need to set our own priorities according to the local context and local needs. We would be doing very well if we did...So the issue of decentralization is also needed to be looked at, it needs to be considered, and the district be empowered more so that they are able to implement their priorities” *[D_3].

#### The Public

When setting priorities, it is important that the public, as the beneficiaries of the process, are involved. This can be achieved through having public representation on the priority setting committees or in institutions or by conducting public surveys where public opinions are considered during the priority setting process. The documents reviewed showed that there are efforts to include representatives from the public on various committees, such as the NITAG, that set priorities for new vaccines. The public is also represented through civil society organizations that participate in the HSSIP processes. This study was unable to assess *actual* participation of the public at these fora. In addition, the document review shows that, community level studies were conducted before the introduction of HPV and PCV. However their purpose was to assess the public’s readiness and their perceptions about the vaccines.^16-18^

#### Use of a Clear Priority Setting Process/Tool/Method

According to the framework, priority setting should involve the use of an explicit process, tool or method. The documents reviewed showed that Uganda’s MoH does not use an explicit method or framework for priority setting, although they have articulated a clear process. Despite the existence of a clear process, it is not always followed. For example, in 2008, yellow fever and hepatitis B vaccines were identified as priorities in HSSIP II,^9^ while the CMYP^11^ identified and prioritized the introduction of HPV vaccine in 2008 and increased surveillance of hepatitis B and rotavirus in 2008 and 2006 respectively. However, no mention was made of yellow fever vaccine. In HSSIP III,^8^ the focus was on Haemophilus influenzae type B, pneumococcus, yellow fever, hepatitis B and rotavirus vaccines, and there was no mention made of HPV. However, in the corresponding CMYP,^12^ the priorities were to complete the programmatic evaluation of HPV vaccination by 2010, to introduce PCV into the routine immunization schedule by 2010, and to vaccinate 90% of children with PCV3 by 2013. In addition, the CMYP aimed to introduce Rotavirus vaccine into the routine immunization schedule by 2013.^12^

#### Use of Evidence

##### Pneumococcal Conjugate Vaccine

According to Kapiriri and Martin’s framework,^6^ using evidence to guide priority setting can discourage the use of controversial criteria, such as politics, hence increasing the appropriateness of decision-making. The review of GAVI applications also showed that the MoH placed considerable emphasis on generating locally-relevant data to support the introduction of the PCV vaccine. For example, a sentinel disease surveillance and reporting system for pneumococcal disease was initiated in 2002, which generated evidence that supported the case for the introduction of PCV. Evidence on the high disease burden of invasive pneumococcal diseases such as meningitis and pneumonia (respectively 28/1000 and 3212/1000 for children below 5 years), the anticipated benefits, especially for children, and the health system’s readiness, as demonstrated by the EVMA, all supported the introduction of PCV.^13^ In addition to this, several studies were conducted prior to the introduction of PCV, for example, a cost-benefit analysis study^18^ and another study exploring the role of the public.^19^

The evidence from the Ugandan surveillance system supported the selection of PCV-13, which addresses more strains of pneumococcus and is more compatible with the Ugandan context, in terms of cost, ease of handling, management, storage, and fit in the immunization schedule; however, excessive demand for PCV-13 at the global level led to the MoH being forced to choose PCV-10 instead of the more locally appropriate PCV-13.

##### Human Papilloma Virus Vaccine

Several studies were conducted and published prior to the introduction of HPV vaccine in Uganda. Some of these studies examined opportunities and obstacles in delivering the vaccine^17,20^ formative research by PATH^21^ and research on delivery and readiness.^16,22^ However, it is not clear if these studies were conducted in preparation for the implementation of the HPV vaccine. They may have not influenced the initial prioritization of the vaccine.

#### Use of Explicit Relevant Priority Setting Criteria

In order for priority setting to be fair, it should be based on relevant reasons or information such as epidemiological information, considerations of equity, or data on costs and effectiveness of interventions. Discussions with participants and the HSSIPs indicate that acceptable criteria, such as use of evidence, and considerations of equity, in addition to unacceptable criteria, such as political sentiment, and industry and donor priorities influenced the decision to prioritize the HPV and PCV vaccines.

##### Pneumococcal Conjugate Vaccine

Our interviews with study participants and a review of documents reveals that considerations of equity, (since PCV focuses on children who are considered to be vulnerable), cost effectiveness, and burden of disease were critical in the MoH’s choice to introduce PCV. For example, one respondent explained that based on cost effectiveness, PCV was planned to be introduced ahead of vaccines such as HPV.

##### Human Papilloma Virus Vaccine

Unlike PCV, there is limited information on the criteria that facilitated the MoH’s prioritization of the HPV vaccine. Participants described how equity was an important criterion considered in the prioritization of HPV. According to one Ugandan policymaker, the HPV vaccine targets women, and its prioritization was a result of the importance of gender equity. He explained that the MoH’s prioritization of the HPV vaccine is a good example of the way in which equity as a criterion can trump other considerations such as evidence of burden of disease:

“*I think HPV was likely taken on for equity reasons…Women felt they were being ignored…if we were to set priorities based on morbidity patterns [alone], I think HPV wouldn’t have appeared”* [U_ 9].

Some participants identified criteria that they thought not to be relevant to prioritizing new vaccines. For example, global participants argued that HPV became a priority because pharmaceutical companies promoted the vaccine, while national respondents thought it was partly influenced by political sentiments. According to one participant, industries are more likely to promote the introduction of more profitable vaccines like HPV compared to vaccines for diseases such as measles:

*“…if there is a donor with a lot of money and he has a new product… they will find a way. We are challenged at this moment with the HPV…I think it’s a fantastic vaccine and every woman should have it…but industry is so interested in selling that and they’re not so interested in selling the 23 cents for measles vaccine.…usually [it is] the governments or the research scientists pushing [their priorities] but now we have the industry actually who can make a profit out of these things pushing their priorities…”* [G_8].

Some of the national-level participants argued that the government rushed to adopt the HPV vaccine, despite the fact that this decision was not necessarily a result of evidence or logistical considerations. As one researcher from Uganda explains, the influence of politics can offset priorities based on evidence, and can derail considerations of the impact of adopting a new vaccine on the health system:

*“…But a lot of times what I’ve seen is it’s often a political decision. So there’s a difference between the technical and the political priorities. Of course for a politician the disease that they see more happens to be cancer so there was a big push [to prioritize the] HPV vaccine even before PCV and rotavirus vaccines. So that’s a big challenge. If tomorrow you told [the President] that there’s now a malaria vaccine that will be ready, you have to go buy this vaccine. Because immunization is one of the areas that are very important to him, [it will be done without consideration of] the technicalities of introducing that new vaccine, the cold chain requirements, how are they imported, is there enough money?”* [U_13].

#### Publicity of Priorities and Criteria

Transparency is important to fair decision-making. The publicity condition requires that the decisions and the rationales and criteria on which they are based are available to the public. In Uganda, attempts to publicize decisions occur mainly through the print media. There were a couple of media reports on the introduction of the new vaccines, with varied content. For example, newspapers reported on the incidence of meningitis and cervical cancer, the government’s plans, with support from GAVI, to introduce PCV,^23^ and the HPV pilot implementation.^21^ Furthermore, there were media reports about the magnitude of the health problems being addressed and the need for the public to respond positively.^24-26^ The launches of vaccines in many districts (for example, the launch of PCV by the president) were also often highly publicized.^23^ Other media reports were about the limitations of the new vaccine programs with some questioning the sustainability of the funding (given the prior misappropriation of funds), and history of vaccine stock outs.^29-32^ In some of the newspaper reports, the public reacted negatively to HPV. Although the decision to adopt both vaccines was disseminated to the public through popular media, these media reports did not inform the public of the process and rationale used to identify the vaccine priorities.

#### Functional Mechanisms for Appealing and Revising the Decisions

According to this condition, there should be mechanisms for appealing the decisions and opportunities for revising the decisions, based on new evidence. This study did not identify any explicit appeals and revisions mechanisms. The only avenue for channeling complaints seems to be through the media (discussed above).

#### Functional Mechanisms for Enforcement

According to Kapiriri and Martin’s framework,^6^ there should be internal or external mechanisms to ensure a fair process. While the MoH does have structures that oversee the implementation of the new vaccines, there were no mechanisms that explicitly focused on assuring that the 4 conditions of a fair process are met.

#### Fairer Priority Setting Processes

According to the framework, priority setting should be perceived as fair. A fair process meets the following conditions: relevance – ie, stakeholder participation, use of evidence, use of explicit relevant criteria – publicity, revisions and enforcement (see Table 4).^33^ From the above discussion it is clear that while there were attempts to meet the conditions of a fair process, none was fully met.

#### Increased Public Confidence in and Acceptance of Decisions

As discussed above, newspaper reports suggested that the public reacted negatively to the introduction of the HPV vaccine. However, no explicit complaints or revisions to the decisions were documented or mentioned by respondents.

#### Increased Stakeholder Understanding, Satisfaction and Compliance With the Priority Setting Process and Decreased Dissentions

Stakeholders are key to the success of any priority setting process. Stakeholder involvement in the process or their exposure to the publicized information (discussed above) would facilitate their understanding of priority setting. Their involvement would also contribute to their satisfaction and compliance with the outcome of the process and would result in reduced dissentions with the decisions. While stakeholders seemed satisfied with the documented “ideal” process, there was dissatisfaction with the actual process, especially in relationship to the prioritization of HPV in 2005-2010 and 2010-2015 in the CMYPs since the actual process did not match the HSSIP. Informants’ comments suggest that the process of setting and implementing priorities was not inclusive, and that there was decreased understanding and satisfaction with the process, which resulted in apathy in implementing priorities, especially within the districts. While stakeholders reported dissatisfaction, no formal complaints were reported.

#### Improved Internal Accountability/Reduced Corruption

Accountability ensures that stakeholders gain confidence in the priority setting process and the priority setting institution. This confidence will increase stakeholders’ compliance and hence facilitate more appropriate use of resources, augmenting successful priority setting. Interviews with stakeholders from the MoH revealed that although stakeholders were aware of the institutions’ existing priority-setting process, there was limited transparency and understanding of the actual prioritization process in the case of HPV.

#### Strengthening of the Priority Setting Institution and Impact on Institutional Goals and Objectives

In some instances the priority setting institution may be independent of the MoH, however, in the case of priority setting for vaccines, the MoH is responsible; therefore, the parameters related to the priority setting institution are assessed under the health system parameters.

#### Impact on Health Policy and Practice

The priority setting process for the new vaccines did not impact health policy. However, the implementation of the new vaccines did change health practice since at the time of the study, PCV had already been approved for integration into the routine vaccination schedule and there were plans to do the same for HPV.

#### Achievement of Health System Goals

Successful priority setting should have a positive impact on the health system’s achievement of its goals, which include improving population health. The effectiveness of PCV and HPV vaccines is well documented. While it was not possible to objectively assess the impact that the vaccines have had on alleviating the disease burden, the population coverage of the vaccines can be used an indicator for their potential impact. At the time of the study however, HPV had not been rolled out in the whole country.

#### Improved Financial and Political Accountability

Financial accountability relates to improved tracking and reporting on financial allocations, disbursement and utilization of financial resources, while political accountability relates to the degree of integration of public interests. One respondent from the MoH argued that donor influence, combined with weak leadership and lack of financial accountability on the part of those working in the MoH had resulted in a situation in which most of the resources for immunization are externally controlled, which impacts timely disbursement and implementation.

*“…of course we had these other challenges of, what can I say…corruption or accountability issues. So that has meant that…they are resources in the hands of partners for whom they [the government] doesn’t have control. So I think that limits a lot of capacity of the government to implement…people in the Ministry could be described as can I say beggars?… They are dependent on partners…so…if I give an example for immunization, we’ve done a cost evaluation and found that most of the resources for immunization are lying outside the control of the Program of Immunization. They are either with a big entity, National Medical Stores, or they are elusive or they are quite ear-marked because they are GAVI funds…you can only spend on this…”* [U_10].

The above sentiments were also alluded to in the newspaper reviews where delays in district accountabilities for GAVI funds for PCV introduction reportedly resulted in delays in disbursements of other funds. This led to delays in key activities necessary to prepare the health system for PCV introduction, particularly health worker training. In turn, there were delays in the final roll out of the vaccine, even when it had already been launched.^32^

#### Increased Investment in the Health Sector and Strengthening of the Healthcare System

For the organizations that invest in health systems in LICs, increased financial accountability often builds their trust and increases their investments into the health system. Furthermore, a successful priority setting process should facilitate the strengthening of the priority setting institutions. In turn, strong institutions are likely to implement priority setting successfully.

According to the interviewees, the priority setting process resulted in increased investment in the health sector through the introduction of GAVI funding to support the MoH’s delivery of HPV and PCV. The introduction of the new vaccines resulted in the training of health personnel, strengthening the cold chain and strengthening the disease surveillance system. However, these resources and activities were directed narrowly towards vaccines.

GAVI funding to support the MoH’s delivery of HPV and PCV increased investment in the health sector. However, respondents argued that development assistance partners’ influence on the prioritization of new vaccines was problematic, because of the hidden costs to national health systems associated with the so-called free vaccines. They emphasized the considerable work and resources from the health system required to introduce new vaccines such as HPV; in addition to co-financing, participants discussed additional costs, such as the cold chain infrastructure and training for health workers required. For example, a participant argued that in the past, the influence of development assistance partners was less problematic since vaccines were always a “good buy.” However, newer vaccines such as HPV are more costly. This means that it is important for development assistance partners to support countries to make decisions based on national preferences and good data.

*“…So if you’re measuring progress then what the people [development assistance partners] that are being measured are going to do is they’re going to run around and try to force countries to introduce HPV because then they look good. I said you know you’re undermining national decision-making by setting a goal in that fashion…So you’re absolutely right there are incentives in place to you know push countries to make poor decisions…until very recently and one of the challenges the vaccines are going to face in the very near future is that vaccines were always a good buy... but what’s happening and is going to happen in the very near future and what GAVI has to be aware of is the new vaccines are not as cost effective…”* [G_5].

#### Contextual Factors

Although the MoH had introduced the 2 vaccines, participants identified contextual issues that were reported to have weakened the institutional capacity of the MoH. These negatively impacted the priority setting process for both vaccine.

Participants identified a number of institutional and resource-related weaknesses in the health system, which impacted institutional capacity and continuity, limiting the MoH’s ability to implement the stated priorities. Respondents from the MoH described how due to apathy and a lack of leadership, priorities stated in the HSSIPs failed to be implemented. A major issue discussed by participants is the negative influence that changes in staffing and leadership at the MoH had on the implementation of HSSIP II and the design of HSSIP III. During the implementation of the most recent HSSIPs, there were multiple changes involving the Ministers of Health and their technical arm, the Director General. New appointments reportedly resulted in poor transitional processes. This inevitably interrupted the implementation of identified priorities. Furthermore, respondents reported that some of the new leaders seemed anxious to introduce new initiatives, while others were not knowledgeable about the previous HSSIP.

Respondents also discussed the negative impact of a lack of resources on the institutional capacity for the implementation of vaccine priorities. Participants described 2 seemingly contradictory issues in relationship to the lack of resources at the national level: a lack of resources, and the MoH’s inability to use existing resources wisely. While resources may be available at the national level, district respondents reported that the disbursement of funds from the Ministry to districts for the implementation of priorities was often delayed considerably, which negatively affected the implementation of priorities. With regard to the latter, one district-level participant describes:

*“…First of all our failure in most cases comes first out of failure to have the necessary funds to be disbursed from the Ministry, to the district. In most cases we may plan that we expect 5 billion from the MoH and then we realize it…has been cut…by the Minister of Finance, so that already affects the financial disbursement. Secondly, at times the funds are not sent in time, and it affects how events are planned”* [D_1].

MoH respondents also described how limited resources negatively influenced districts’ delivery of the new vaccines because they impacted key supports for implementation. For instance, one respondent explained that a lack of funding had resulted in a drop in staffing in the MoH planning department, which is responsible for supportive supervision for the district. As a result, there was far less interfacing with the district. For 2 years, no area teams existed, which are the primary structures for communication with the districts. A lack of staffing meant that the dissemination of the implementation plans for the new vaccines were not well actualized at the district level.

Another major challenge discussed by participants is a stagnant budget for vaccines. Respondents described how the budget for routine vaccines has not grown, however, the number and cost of new vaccines such as HPV and PCV is expanding, in addition to the population size, number of districts and the disease burden, which increases the pressure on the national budget. As one participant noted:

“…*But overall the budget for the health sector over the last few years has actually not grown…actually it has been going down and yet we have seen population but also disease burden…not just burden but the case mix of diseases has been expanding but also …the packages have been expanding but not necessarily the resources”* [U_18].

To ameliorate these issues, global level respondents suggested that development assistance partners should align their priorities with those of national governments. However, as one technical partner argued, while the concept of donor harmonization has been promoted, it has not been well operationalized; the harmonization of the stakeholders involved in vaccine priority setting is negatively impacted by weak institutional capacity in countries such as Uganda:

*“…harmonization means that everybody agrees that we need to do things differently. Partners should align among themselves in the first place and secondly they should align with countries’ priority. But it is still a challenge to…to operationalize this approach at country level and I think it is because you know the system in most of these countries is still weak…So each partner will then go, you know, their own way because it is just difficult for the national health system to coordinate all this. And also the partners want quick results and if there isn’t results coming very quickly then they will do the things by themselves. So the way I think, what will help to…really sort out this is to have strong system and institutions in the countries…”* [G_6].

## Discussion

This paper contributes to the current literature by describing and evaluating priority setting for 2 new vaccines in Uganda using Kapiriri and Martin’s framework,^6^ which was developed to evaluate priority setting in these contexts. To our knowledge, this is one of the first case studies to examine the influence of the global, national and sub-national levels on priority setting for new vaccines. While this study draws on the Ugandan context, the national and local decision-making dynamics in the Ugandan public health system, as well as the economic and political power differences between its MoH and development assistance partners, are similar to those in other LICs, and thus, provide a basis for generalizations beyond the Ugandan context.

The parameters of successful priority setting are summarized in Table 5. First, the parameters that could be assessed and whether or not the priority setting processes satisfied these quality indicators is discussed. Lastly, the parameters that could not be assessed and the implications of these findings are discussed (see Table 4).

Based on the parameters in Kapiriri and Martin’s framework,^6^ the process for prioritizing both vaccines was successful in a few ways. First, although no framework was used in the priority setting process for either vaccine, our data suggest that the decisions to introduce both HPV and PCV were based on considerations of evidence; while HPV used evidence from a pilot project in 2 districts, PCV used evidence from sentinel surveillance reports and community studies. In addition, both vaccines were prioritized based on explicit relevant criteria: cost-effectiveness for PCV and burden and disease and equity for HPV. While stakeholders from the MoH were generally satisfied with the decision to introduce PCV, several expressed dissatisfaction with the prioritization of HPV in 2005-2010 and 2010-2015 in the CMYPs since the actual process did not match the HSSIP. Stakeholders’ understanding of the priority setting process for PCV was better as compared to HPV since PCV was one of the original priorities set by the MoH, while HPV was not. In the case of HPV, the allocation of resources toward the vaccine did not successfully follow the original plan set by the MoH.

According to some respondents, unacceptable criteria such as external monies and influence affected priority setting in 2005-2010 and 2010-2015. The prioritization of HPV was based more on the influence of donors and industry and political sentiment than technical priorities; this contributed to stakeholders’ dissatisfaction with the priority setting process. Banura and colleagues^16^ describe how both formulations of HPV vaccines were introduced to Uganda through donations to the MoH. First, GlaxoSmithKline donated 50 000 doses of Cervarix to vaccinate girls in Nakasongola and Ibanda districts. Then, Merk and Co. donated 1600 doses of Gardasil to vaccinate 500 HIV-positive girls.^13^

A lack of participation of key stakeholders contributed to dissatisfaction with the priority setting process for both HPV and PCV. There was a sense that because of a lack of leadership in the MoH, compliance with the priority setting process had decreased and the process had become less consultative, in which the participation of key actors such as MoH staff and the district officers had declined. This was in part explained by the changes in top leadership in the MoH and a reduction in development assistance partners’ adherence to a consultative priority setting process. However, despite the dissatisfaction with the priority setting process expressed by many of our respondents in interviews, no dissentions were formally recorded in reports. In some of the newspaper reports, the public reacted negatively to the new vaccine (HPV) but no explicit complaints or revisions to the decisions were documented or mentioned by our respondents. This could be explained by the lack of explicit mechanisms for appealing the decisions.

In terms of the achievement of health system objectives, indicators such as the percent reduction in disability-adjusted life years* (*DALYs*)* could not be measured; however at the time of the data collection, the implementation of both vaccines was contributing to the health system’s goals of reducing morbidity. Arguably, the implementation of PCV, which was scaled up completely across the country, contributed more to this goal than HPV, which remained at the pilot phase at the time of the study. In terms of increasing investment into and strengthening of the health sector, the priority setting process may have met this parameter; this is because the introduction of the new vaccines involved substantial investment into the health sector and supported other health sector activities such as disease surveillance and capacity building. However, this was specific to EPI.

While GAVI expects and facilitates improved financial accountability at the national level,^15^ this did not seem to trickle down to the districts where delays in district accountabilities for PCV affected vaccine implementation; although this problem was later rectified.

Consistent with the literature, contextual factors impacted the parameters of successful priority setting.^34^ Some of the factors, such as strong political commitment to vaccines facilitated the introduction of the new vaccines. However, most of the contextual factors negatively impacted priority setting. For example, the political decisions to change the top management, the influence of donor and industry priorities, and the economic context, which led to a lack of expansion of the budget for the health sector and subsequently resulted in inadequate staff for implementation at the district level, all had a negative influence on priority setting.

A few parameters could not be assessed. The priority setting process should increase public awareness of priority setting, involve the increased input of the public and reflect public values, and facilitate public confidence and acceptance of decisions. The assessment of these criteria was mixed. It was not possible to assess increased public awareness of priority setting and increased public confidence in and acceptance of decisions since these parameters necessitated interviewing members of the public. It was also not possible to assess whether the priority setting process reflected public values, since the documents reviewed do not show the exact role played by the public in the priority setting process. Finally it was also not possible to evaluate the efficiency of the priority setting process and decreased resource wastage, parameters that require real-time assessment.

## Conclusion

Based on the evaluation framework, there are several areas where concerted efforts are necessary if priority setting for new vaccines is to be deemed successful. First, the prioritization of new vaccines should be based on explicit frameworks. The lack of clarity as to why and how HPV was prioritized over other vaccines that had been identified through a national level process calls for commitment to transparency with regards to how new vaccines get onto the political agenda. HSSIPs should be used as the basis for implementing the documented new vaccine priorities.

There is also a need for enforcement mechanisms to ensure that vaccines identified as a priority in the initial HSSIP planning process are not overtaken by vaccines that are not considered to be a top priority. This requires the direct involvement of key stakeholders such as district officers in determining national level priorities. The process should also require public consultations and involvement. This would call for raising public awareness about the priority setting process and the rationale behind the decisions. Monitoring implementation of the new vaccines should also involve monitoring the public response to the set priorities and their implementation. The existing power imbalances between sub-national governments, national governments and their development assistance partners need to be mitigated by strengthening institutional capacity and leadership; this is essential to ensure that priorities are identified and implemented through an inclusive, transparent, and efficient process.

To a great degree, Kapiriri and Martin’s framework and sources of information^6^ provided viable guidance for evaluating the degree to which the priority setting processes for identifying new vaccines worked in Uganda. The results re-emphasize the need for evaluation to be built in the priority setting process a priori, and that information on priority setting is gathered throughout the implementation cycle as opposed to the traditional ex-ante evaluations.^5^

## Acknowledgements

This study was funded by the Canadian Institutes for Health Research (CIHR), Ottawa, ON, Canada. We acknowledge the contributions made by our research assistants and the study participants.

## Ethical issues

This study was reviewed and approved by the McMaster Research Ethics Board and Makerere University School of Public Health Research Ethics Board. Each respondent signed a consent form prior to participating.

## Competing interests

Authors declare that they have no competing interests.

## Authors’ contributions

LK conceptualized the study, participated in data collection, analysis and conceptualizing and developing the manuscript. LW participated in data analysis, conceptualizing and developing the manuscript.

## Authors’ affiliations

^1^Department of Anthropology, McMaster University, Hamilton, ON, Canada. ^2^Department of Health, Aging and Society, McMaster University, Hamilton, ON, Canada.

## Key messages

**Implications for policy makers**

- An increasing number of new vaccines on the market and limited resources will always necessitate policy-makers to make informed decisions about which vaccines to introduce first.
- Using the examples of the prioritization of human papilloma virus (HPV) and the pneumococcal conjugate vaccine (PCV), Kapiriri and Martin’s framework was able to identify areas of good practice and areas that require improvement.
- There is a need to integrate real-time evaluation into the priority setting processes for new vaccines such that information is gathered throughout the implementation cycle rather than ex-ante.

**Implications for public**

New vaccines have the potential to improve quality of life, especially for those living in low-income countries (LICs). However, the number of new vaccines that LICs can potentially adopt is often greater than what LICs and their development assistance partners can afford to introduce. For this reason, LICs need to follow a priority setting process when identifying which vaccines to introduce first. Recommendations to improve priority setting for new vaccines should be based on an understanding of how these decisions are currently made. This study evaluates the decision-making processes that led to the introduction of two new vaccines – PCV and HPV – from the global level to the point of delivery in Uganda. While the decision-making process was appropriate in some areas, it also fell short in others; for instance, there was a lack of publicity about the need to set priorities and the rationales behind the priorities that were identified. Transparency requires that decisions and their rationales are available to the public.
